# Cocaine Induces Cytoskeletal Changes in Cardiac Myocytes: Implications for Cardiac Morphology

**DOI:** 10.3390/ijms22052263

**Published:** 2021-02-24

**Authors:** Avnish Verma, Ayse Orme Merve, Vytautas Remeškevičius, Pola Sobiecka, Luke Taylor, Scott Lawton, Ben P Jones, Elena Polycarpou, Jason Bennett, Brian Rooney

**Affiliations:** 1 Cardiovascular Science, Institute of Life Course and Medical Sciences, Faculty of Health & Life Sciences, University of Liverpool, Liverpool L7 8TX, UK; avnish.verma@liverpool.ac.uk; 2 School of Life Sciences, Pharmacy and Chemistry, Kingston University, Penrhyn Road, Kingston upon Thames, London, KT1 2EE, UK; k1807597@kingston.ac.uk (A.O.M.); V.Remeskevicius@kingston.ac.uk (V.R.); P.Sobiecka@kingston.ac.uk (P.S.); Luke.Taylor@kingston.ac.uk (L.T.); S.P.Lawton@kingston.ac.uk (S.L.); k1314189@kingston.ac.uk (B.P.J.); E.Polycarpou@kingston.ac.uk (E.P.); 3 Centre for Sport, Exercise and Life Sciences (CSELS), Coventry University, Pharmacology and Therapeutics, Alison Gingell Building, Whitefriars Street, Coventry CV1 2DS, UK; ad2619@coventry.ac.uk

**Keywords:** cocaine, cardiac myocytes, cytoskeleton, actin rearrangement, cardiac toxicity

## Abstract

Cocaine is one of the most widely abused illicit drugs worldwide and has long been recognised as an agent of cardiac dysfunction in numerous cases of drug overdose. Cocaine has previously been shown to up-regulate cytoskeletal rearrangements and morphological changes in numerous tissues; however, previous literature observes such changes primarily in clinical case reports and addiction studies. An investigation into the fundamental cytoskeletal parameters of migration, adhesion and proliferation were studied to determine the cytoskeletal and cytotoxic basis of cocaine in cardiac cells. Treatment of cardiac myocytes with cocaine increased cell migration and adhesion (*p* < 0.05), with no effect on cell proliferation, except with higher doses eliciting (1–10 μg/mL) its diminution and increase in cell death. Cocaine downregulated phosphorylation of cofilin, decreased expression of adhesion modulators (integrin-β3) and increased expression of ezirin within three hours of 1 μg/mL treatments. These functional responses were associated with changes in cellular morphology, including alterations in membrane stability and a stellate-like phenotype with less compaction between cells. Higher dose treatments of cocaine (5–10 μg/mL) were associated with significant cardiomyocyte cell death (*p* < 0.05) and loss of cellular architecture. These results highlight the importance of cocaine in mediating cardiomyocyte function and cytotoxicity associated with the possible loss of intercellular contacts required to maintain normal cell viability, with implications for cardiotoxicity relating to hypertrophy and fibrogenesis.

## 1. Introduction

Cocaine is a commonly abused recreational drug, due to its stimulant and euphoric activities in the brain and central nervous system [1]. Owing to its vasoconstrictive properties, cocaine has long been regarded as an agent for cardiac dysregulation attributed to arterial spasm mediated ventricular fibrillation and cardiac arrest. The antiarrhythmic properties of cocaine stimulated by blocking sodium and potassium channels forms the basis of several cardiovascular implications; most notably, pro-thrombosis and intracoronary thrombosis mediating myocardial infarction, inflammation involved in cardiac pathophysiology and angina secondary to these complications [2]. In emergency department settings, angina and myocardial infarction are the two most common cardiac complications associated with cocaine abuse, accounting for 57% and 77% of emergency cases respectively, with the highest risk occurring within the first 60 min following cocaine abuse [3]. A case-crossover study reported that the onset of myocardial infarction was 24 times above baseline in the 60 min following cocaine use. A 2005 study suggested that up to 84% of patients reported angina induced by cocaine, with 43% of patients reporting abnormal ECG traces [3]. Furthermore, cocaine toxicity has been shown to be a regulating factor of dilated cardiomyopathy owing to cardiac architectural alterations associated with myofibril destruction and interstitial fibrosis [4]. Using cardiovascular magnetic resonance (CMR) technology for the evaluation of ventricular metrics, Maceira et al. [4] highlighted that 71% of patients reporting long term cocaine abuse were clinically/histologically assessed with cardiovascular disease relating to enhanced left ventricular mass, localised fibrosis, reductions in ventricular systolic volume, and ventricular ejection fractions, with 30% of the cohort examined being identified with myocardial damage. 

Cocaine’s role in mediating structural changes in the cytoskeleton has long been associated with the pathogenesis of addiction. Numerous studies have identified cocaine as a potent regulator of actin cytoskeletal dynamics in neuronal cells such as in the nucleus accumbens (NAc) and have suggested that the structural remodelling of these tissues is the pathophysiological basis of addiction [5]. Despite research linking cocaine to the modulation of the actin cytoskeleton and architectural alteration of cardiac tissue, the mechanism and extent of cardiac tissue remodelling remains unclear. Cardiac fibrosis following intermittent myocardial infarctions represents a common pathological implication of chronic cocaine abuse. A recent study by Lee et al. [6] demonstrated reductions in cardiac fibrosis following oestradial treatment, with this abrogation reported to be mediated by the downregulation of Rho-ROCK signalling, cofilin phosphorylation and F:G actin ratio, demonstrating the importance of the Rho/ROCK/LIMK pathway and actin reorganisation in cytoskeletal mechanisms of fibrogenesis. Supporting this, Pereira et al. [7] examined the role of cocaine in vascular-endothelial dysfunction and reported the enhancement of leukocytic Rho-kinase activity in rat vascular walls, in vitro vascular cell ROCK activity and enhanced platelet adhesion upon treatment with plasma from cocaine abusers. Furthermore, a study by Pradhan et al. [8] demonstrated that enhanced nitric oxide, endothelin-1 production, and in vitro cocaine exposure dependent monocyte adhesion potentiates the role of cocaine in regulating pathological development. 

Despite numerous studies linking cocaine to the modulation of the actin cytoskeleton and remodelling of cardiac tissue, most of the research in this area is representative of retrospective or autopsy studies that have discussed cocaine’s involvement in dysregulated cardiovascular functioning. Few studies have investigated the in vitro effect of cocaine in cardiac cells or determined the signalling events in relation to detrimental tissue remodelling. This study investigated surface and internal cytoskeletal regulators that are modulated by cocaine and the subsequent changes in cellular phenotype. In addition, we examined if cocaine’s effect on cardiomyocytes is governed by a spectrum of treatments that regulate functional responses and cytotoxicity.

## 2. Results

### 2.1. Cocaine Elicits a Dose Dependent Response on Cell Viability and Morphology

**Figure 1:**
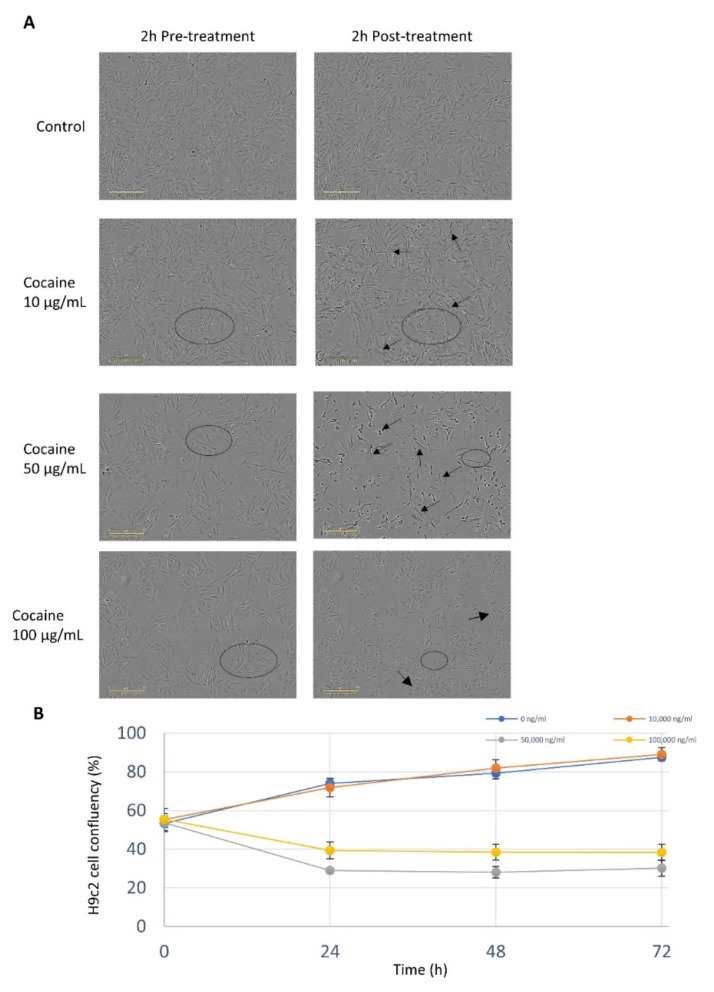
H9c2 cardiomyocyte morphology and cytotoxicity following cocaine exposure. (**A**) H9c2 cardiomyocytes indicated prominent stress features, including loss of compaction and cell—cell integrity after two-hour exposure at doses of 10–100 μg/m (black circle and arrows, respectively). Images are representative of three independent experiments and were captured using IncuCyte Zoom® Live cell analyser (scale bar 300 µm). (**B**) H9c2 cells treated with ranging low-to-high cocaine dose concentrations for 72 h without drug replacement. Data represents mean values ± SEM (*n* = 24, for each data point on a 96—well transparent culture plate recorded from three independent experiments). Statistical comparison was performed by a one-way ANOVA followed by the post hoc Dunnent’s comparison test, * *p* < 0.05 for each group vs control.

H9c2 cardiomyocytes treated with 10 µg/mL of cocaine demonstrated no change in cell viability, however, treatments at concentrations between 50 µg/mL and 100 µg/mL significantly (*p* < 0.05) reduced cardiomyocyte cell viability and proliferation (Figure 1A,B). The growth kinetics for cardiomyocytes treated with cocaine concentrations of 10 µg/mL were not significantly (*p* = 0.999) altered compared to the control (Figure 1). Gross morphological changes in response to cocaine were also studied; H9c2 cardiomyocytes exposed to low cocaine concentrations (1–10 µg/mL) maintained proliferative capacity and morphological integrity. Treatments of 10 µg/mL demonstrated enhanced cell spreading and size, with higher concentrations (50 µg/mL) resulting in gross morphological changes, diminution of cellular integrity, individual cellular compactness, and enhanced membrane protrusions (Figure 1A). Interestingly, 100 µg/mL cocaine treatments did not mediate the formation of membrane extensions but instead resulted in the loss of cytoplasmic integrity. These results report a dose-specific relationship between H9c2 cardiomyocyte cell viability and cocaine exposure. 

### 2.2. Cocaine Mediates Increased Cell Migration and Wound Closure

**Figure 2:**
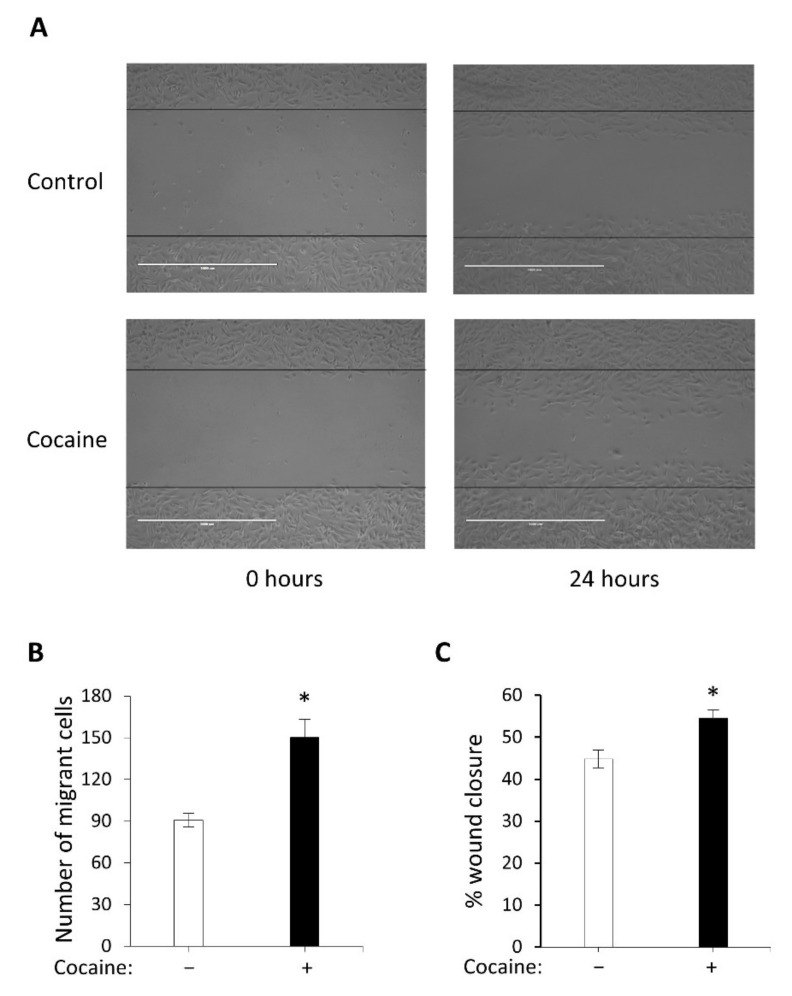
H9c2 cardiomyocyte migration following cocaine treatment. (**A**) H9c2 cardiomyocytes were exposed to complete growth medium or 1 μg/mL cocaine treatments for 24 h. (**B**) Migrant cells were quantified by Image J analysis and compared against non-treated cells. (**C**) Cell wound penetration levels were determined manually by equivalence of the scale bar and compared against non-treated cells. All data represent mean values ± SEM of three independent experiments (*n* = 6, for each data group on a six-well transparent culture plate). Statistical comparison was performed by a two-tailed Students unpaired t-test, *p < 0.05 for treated *vs* control. Images representative of three independent experiments (scale bar 1000 µm).

Cocaine treatments of 1 µg/mL stimulated increased cell motility into the artificial wound within a 24-h period. H9c2 cardiomyocyte migration was significantly enhanced (*p* < 0.05) compared to control (Figure 2). Stimulated wound migration relies upon cell-cell communication and depth of penetration into the wounded area. Our results indicate that cells counted within the wound following treatment with cocaine, resulted in a significant increase (*p* < 0.05) in percentage wound closure compared to the control (Figure 2B). Our results suggest that exposure to a non-toxic dose of cocaine (1 µg/mL) in H9c2 cardiomyocytes, can mediate increased cell migration and highlights cocaine as a potential modulator for cytoskeletal reorganisation in cardiac cells.

### 2.3. Investigation of Cocaine’s Effect on Cell Adhesion and Proliferation

**Figure 3:**
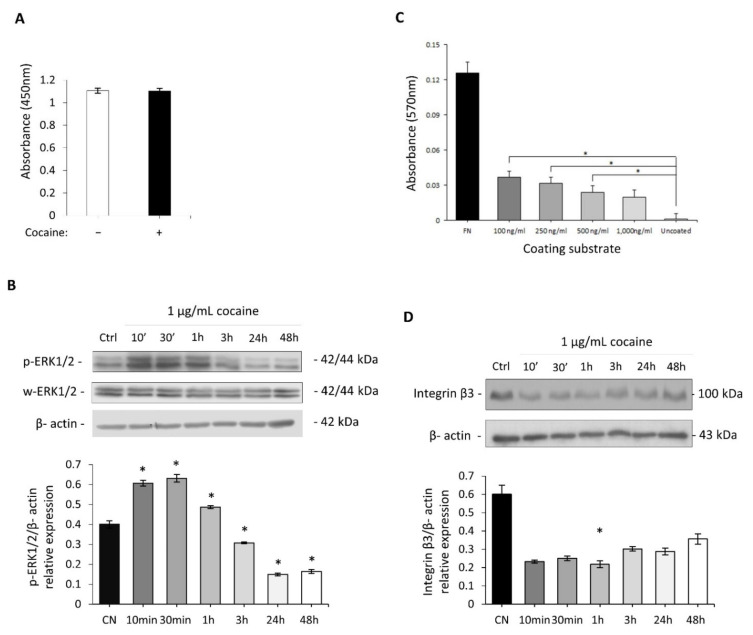
Cell adhesion and proliferation in response to cocaine inH9c2 cardiomyocytes. (**A**) Colorimetric read-out of BrdU incorporation as an indicator of cellular proliferation following 24 h of cocaine treatment. H9c2 cardiomyocytes treated with 1 μg/mL cocaine mediated no change in proliferative capacity following BrDu incorporation vs. untreated control samples. (**B**) Immunoblot analysis of ERK1/2 phosphorylation following cocaine exposure showed initial increase in phosphorylation of ERK1-p44 (Thr-202/Tyr204) and ERK2-p42 (Thr-185/Try-187) within one hour of treatment. (**C**) Absorbance readings for the colorimetric detection of adherent cells following BSA normalisation, with non-treated fibronectin exposed H9c2 cardiomyocytes representing positive controls. Uncoated wells served as negative control. Culture plates were coated with subsequent cocaine concentrations and incubated overnight at 4 °C. Statistical comparison was performed by a one-way ANOVA followed by the *post hoc* Dunnett’s multiple comparison test, * *p* < 0.05. (**D**) Time-dependent protein detection of Integrin-β3 in cardiomyocytes treated with cocaine, show a decrease within 1 h of 1 μg/mL treatment. B-actin served as control. (**B**,**D**) Representative immunoblots of three lysates generated from three independent experiments. Densitometry indicating relative band expression for each blot measured using ImageJ with corresponding statistical analysis performed (One way ANOVA), in all cases *p < 0.05 for treated *vs* control.

To address the possible mechanism relating to the enhanced migratory phenotype observed in the scratch assay and to rule out a possible artefact of enhanced cell proliferation, proliferation studies and the protein expression changes of ERK1/2 at Thr-202/Tyr204 and Thr-185/Try-187 were examined. H9c2 cardiomyocyte cell proliferation was not significantly (*p* = 0.922) altered following 24 h of 1 µg/mL cocaine exposure (Figure 3A). This suggests that the increase in cell migratory potential observed previously (Figure 2) was not governed by an increase in proliferation of cardiomyocytes but instead by increased cytoskeletal rearrangement and migratory function. Cocaine was shown to mediate time dependent increases in expression of ERK1-p44 (Thr-202/Tyr204) and ERK2-p42 (Thr-185/Try-187), with these changes being more pronounced at 10 min, 30 min and 1hr following 1 µg/mL treatment (Figure 3B). Conversely, H9c2 cardiomyocytes exposed to cocaine for 3, 24, and 48 h demonstrated a return of ERK phosphorylation to basal levels. 

We investigated cocaine’s role as an external mediator of cell substrate interactions (Figure 3C). Cocaine treatments of 0.1 µg/mL and 0.25 µg/mL (considered low cocaine doses) mediated significant (*p* = 0.002 and *p* = 0.003, respectively) H9c2 cardiomyocyte-substrate adhesion compared control. Substrate adhesion was also significantly (*p* = 0.029) enhanced in H9c2 cardiomyocytes exposed to 0.5 µg/mL cocaine treatments. Interestingly, the mediation of H9c2 cardiomyocytes adhesion following exposure to 1 µg/mL cocaine treatments was not significantly altered (*p* = 0.093). Also evident is the reduced expression of integrin-β3, a key integrin modulator of cellular adhesion in response to cocaine treatments of 1 µg/mL, this decrease was most prominent within three hours of cocaine treatment (Figure 3D). These results suggest that the cytoskeletal and morphological changes induced by cocaine are dose dependant. 

### 2.4. Cocaine Mediates Changes in Cardiomyocytic Actin Binding Protein Expression

**Figure 4:**
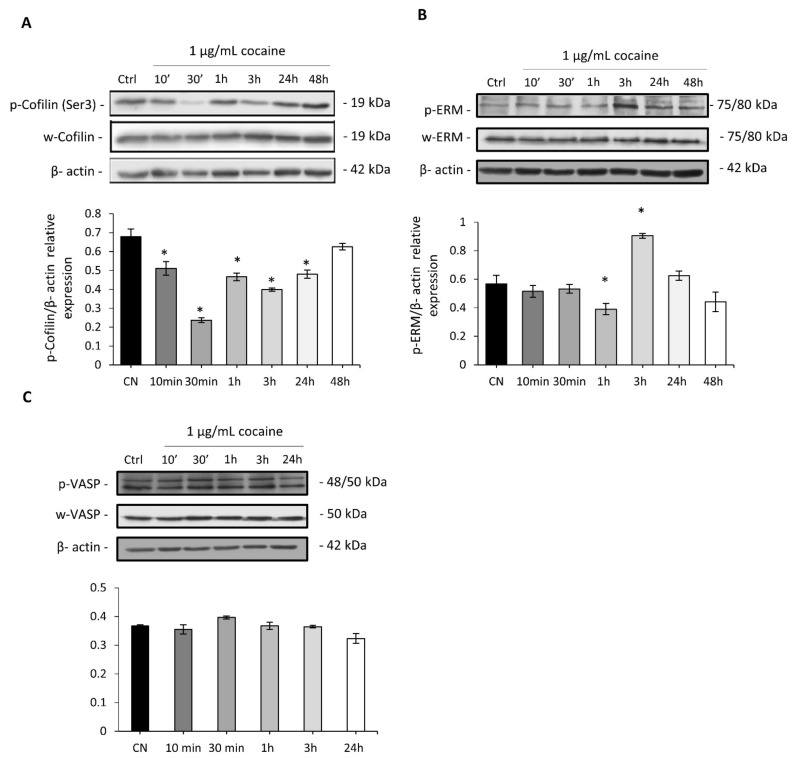
H9c2 cardiomyocyte ABP expressional changes mediated by cocaine exposure without treatment renewal by immunoblotting using ABP specific antibodies. (**A**) Phospho-cofilin (Ser-3) expression changes following short- and long-term exposures regulated several time-point specific changes. (**B**) Phospho-ERM (Thr-567, Thr-564, and Thr-558) expression changes following time course specific cocaine treatments. (**C**) Expression of phospho-VASP at the Ser-239 specific site showed no change to control when treated with cocaine. (**A**–**C**) representative immunoblots of three lysates generated from three independent experiments. Densitometry indicating relative band expression for each blot measured using ImageJ with corresponding statistical analysis performed (One way ANOVA), in all cases *p < 0.05 for treated *vs* control.

Time course treatments with 1 µg/mL of cocaine resulted in expression changes in phospho-cofilin at the regulatory Ser-3 site, most notably decreased phosphorylation, which is associated with increased cell motility (Figure 4A). Investigations into the effects of cocaine on ezrin, radixin and moesin (ERM) and ABPs was also undertaken. ERM was targeted as functional capacity relies upon effective cross- linking between the plasma membrane and the actin cytoskeleton following phosphorylation (activation) at conserved threonine residues (Thr-567, Thr-564, and Thr-558) located within ERMs C’ terminal actin binding domain. Phospho-ERM expression changes in H9c2 cardiomyocytes occurred within three hours of cocaine exposure (Figure 4B). In order to ascertain the full extent of cocaine’s effect on cytoskeletal signal transduction crosstalk, expression changes in vasodilator-stimulated phosphoprotein (VASP) were investigated specifically at its regulatory serine- 239 site (Ser-239), phosphorylation of which impairs F-actin accumulation and filopodia length and number. Time course investigations for protein expression changes in Ser-239 VASP phosphorylation revealed no change following cocaine treatment (Figure 4C). Together, these data suggest the dynamic remodelling of the cytoskeleton in H9c2 cardiomyocytes following cocaine treatments, with changes in key cytoskeletal proteins involved in mediating both direct actin and plasma membrane/actin interactions. 

### 2.5. In-Silico Bioinformatics Docking Studies

**Figure 5:**
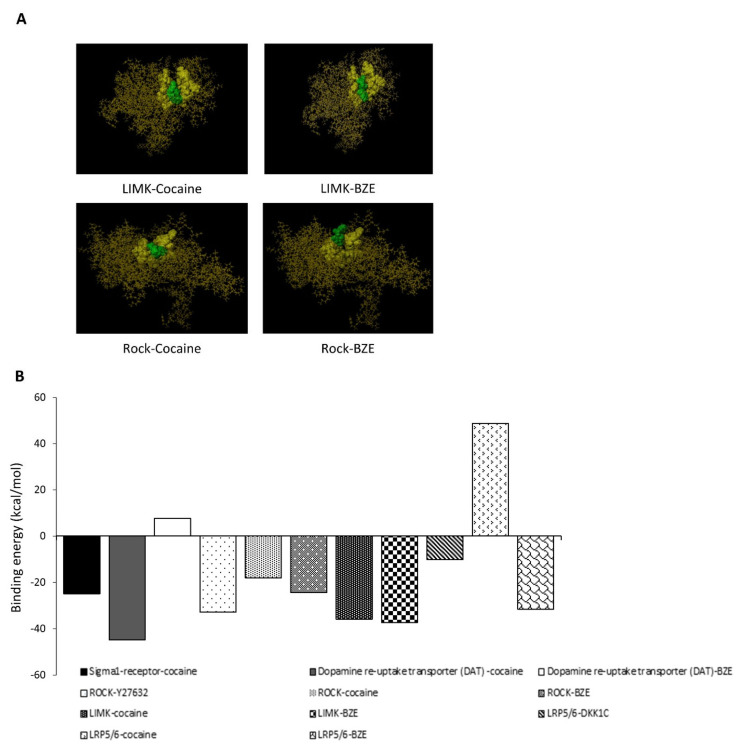
In-silico studies of cocaine and its metabolite BZE modelled against key signalling regulators. (**A**) Schematic of LIM-kinase modelled against cocaine and BZE at the ATP binding site, both ligands indicate similar active site occupancy with BZE representing a greater molecular fit for the specified site of interaction. Yellow and green space filling views represent the LIMK ATP binding pocket and ligand (cocaine and BZE), respectively. Images generated from Scigress docking software. (**B**) Binding energies as measured in kcal/mol generated by in-silico studies of cocaine and its metabolite BZE modelled against key signalling regulators. Each model was generated using acknowledged crystallographic structures and subsequently programmed using the Scigress docking software for targeted interactions. These results suggest that both cocaine and BZE can act as ABP signal transduction mediators, with LIM K and ROCK with lower binding energies comparable to known cocaine receptors such as Sigma 1 and DAT. In relation to canonical Wnt signalling, the LRP5/6 receptor displays high binding energy requirement for cocaine but favourable conditions for BZE.

The possible interaction between cocaine/BZE and modulators of cofilin and cytoskeletal dynamics the Rho-ROCK/LIM-kinase pathway were investigated (Figure 5). In-silico modelling suggested a binding affinity of –32.783 kcal/mol for ROCK/Y-27632, with ROCK-cocaine demonstrating a binding energy of –18.04 kcal/mol at the ATP binding pocket. This infers an input of energy would be required to reach the correct conformations permissive for binding when compared to the same site used by Y-27632 (Figure 5 B). Modelling of BZE against ROCK at the ATP binding pocket suggested a lower binding energy of –24.380 kcal/mol. In addition, to the binding energies identified, the predicated docking conformations suggests similar active site interactions between Y-27632 and BZE, with a seemingly locked and rigid formation for cocaine. Lower binding energies were required for both cocaine and BZE interaction with LIMK, for cocaine and LIMK’s ATP binding site a binding energy of −35.823 kcal/mol for cocaine and −37.344 kcal/mol for BZE was required. These results suggest either direct cell membrane permeation of cocaine or its breakdown prior to interaction with ROCK/LIMK are physiochemically permissible for binding and subsequent modulation of downstream cofilin dynamics. Canonical Wnt/β-catenin signalling and its modulators have been shown to mediate cell migration and differentiation, vascular wall lipid infiltration and cardiovascular diseases associated with hypertrophy and arrhythmias. Modelling cocaine against LRP6 was energetically unfavourable (+48.719 kcal/mol), by contrast BZE generated an affinity of −31.567 kcal/mol, suggesting for the LRP6 co-receptor, BZE is a more prospective mediator for interaction. In summary, the data presented by the in-silico bioinformatics studies highlight cocaine and BZE as possible cytoskeletal and signal transduction mediators with well-known receptors that control these processes and provide a potential mechanism for its modulation of ABP. 

### 2.6. In Vitro Analysis of Cocaine Metabolism in Cardiomyocytes

**Figure 6:**
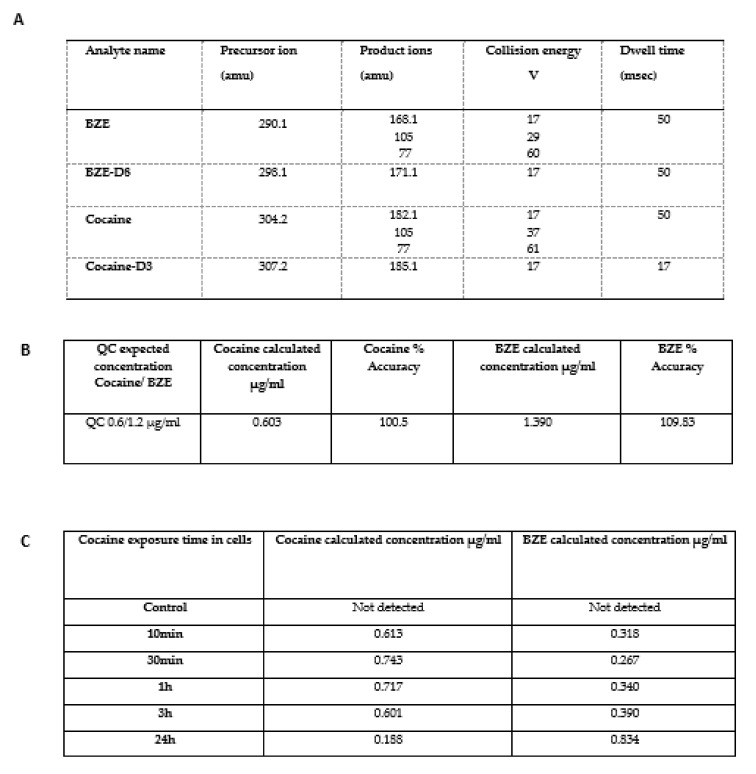
Cardiomyocytes metabolise cocaine to BZE over a 24-h time period. (**A**) a LC-MS method was developed to detect cocaine and BZE in cell media using deuterated internal standards. The ion transitions for each analyte are detailed above. (**B**) In all analysis quality control samples (QC) of extracted spiked media were run alongside samples. These QC demonstrated a calculated concentration accuracy of 90–110%. (**C**) Cells were spiked with 1 ug/mL of cocaine and surrounding media concentration monitored over 24 h. there was a decrease in cocaine concentrations and a concomitant increase in BZE concentration suggesting that metabolism of cocaine to BZE occurs in this model. LC-MS results representative of five extractions generated from five independent experiments.

A novel LC-MS method was developed using the ion transitions listed (Figure 6A), accuracy and precision of the method was demonstrated by running quality control samples extracted from spiked cell media solutions (Figure 6B). Cell media was removed at the time points indicated and subject to sample extraction and LC-MS quantification. Our results indicate that cocaine is metabolised into BZE in Hc92 cardiomyocytes in in vitro conditions. Significantly, there was a decrease in cocaine concentrations with concurrent increase in BZE concentrations over 24 h (Figure 6C). These studies suggest that at longer time points BZE is the dominant substrate present in the cell media and that the effects on cell phenotype, cytoskeletal dynamics, and protein expression post 24 h may be mediated by the biotransformation of cocaine to BZE. 

### 2.7. Cocaine Associated Cellular Microstructural Changes

**Figure 7:**
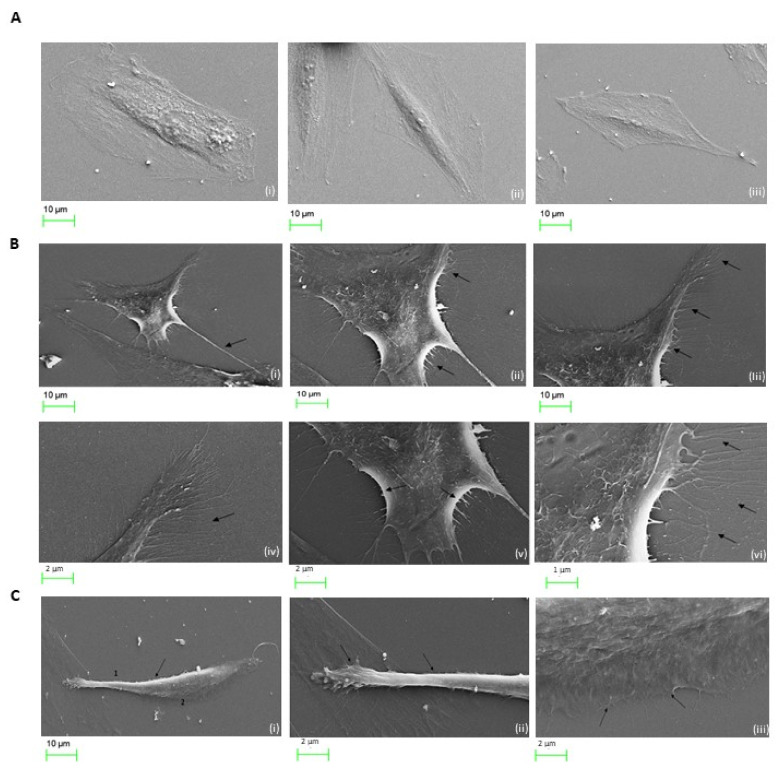
Low dose cocaine treatments result in morphological alterations of H9c-2 cardiomyocytes. (**A**) Untreated H9c2 cardiomyocytes observed under SEM microscopy, normal morphology was retained with intercellular connections and spindle like spreading present at 24 h following culture. Cells display characteristics mono or multi-nucleated spindle to stellate shaped. (**B**) Morphological alterations induced in H9c2 cardiomyocytes following exposure to 1 μg/mL cocaine. Cocaine induced formation of thick and finer membrane extension (i–iv and F–H, arrows, respectively) and folding V arrows). (**C**) Membrane protrusions and cellular attachments in H9c2 cardiomyocytes treated with 1 µg/mL cocaine. H9c2 cardiomyocytes indicated features of altered microstructural architecture, with membrane polarisation (i and ii, arrows), cell-substrate adhesion and retraction observed posterior to sites of membrane folding observed in (i) (iii, arrows). Scale bar for each panel are presented below the respective image with numerical assignment indicating feature specific responses.

**Figure 8:**
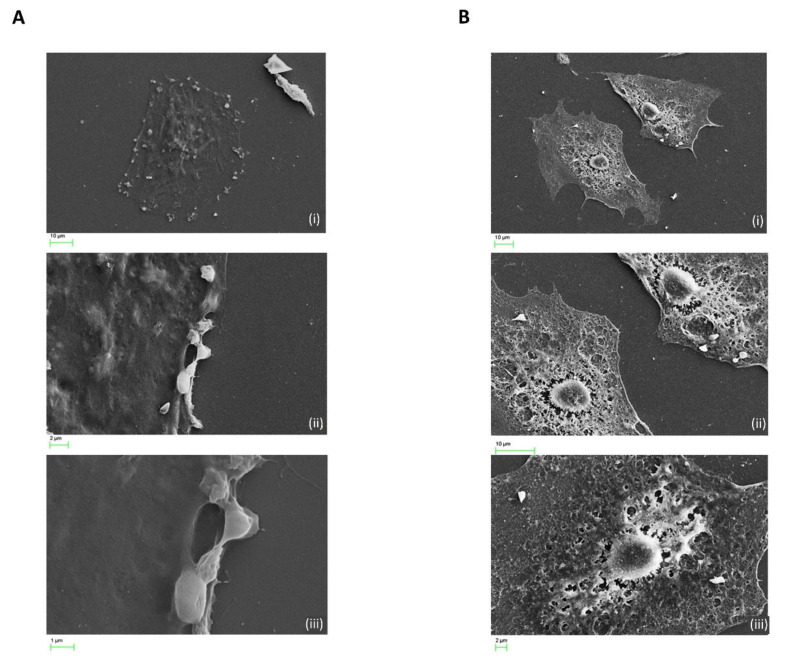
High dosages of cocaine results in microstructural deterioration of cardiomyocytes. (**A**) H9c2 cardiomyocytes treated with 5 μg/mL cocaine. Treatment mediated retraction of cellular outgrowths with significant formation of surface and membrane blebs (i–iii). (iii) Discharge of formed bleb from the cell membrane. (**B**) H9c2 cardiomyocytes treated with 100 µg/mL cocaine. (i–iii) Significant changes associated with injury involving cellular condensation and loss of architectural polarity and intercellular membrane extensions (ii), cell surface collapse, perforation, and cell nuclei separation (ii–iii). Scale bars for each panel are presented below the respective image.

Imaging of untreated H9c2 cardiomyocytes display normal cellular contacts evident of growth and communication as highlighted by intact membrane spanning outgrowths. H9c2 cardiomyocytes exposed to 1 µg/mL cocaine treatments revealed significant morphological alterations compared to control (Figure 7A, Panel i–iii). Cellular compaction resulted in the formation of pronounced membrane extensions associated with condensed membrane folding at protrusion sites (Figure 7B, Panel i–vi). Also evident, was increased dynamic alterations during the exposure periods, with changes in cellular elongation and substrate adhesion indicating morphological expressions acquired for later stages of cellular motility (Figure 7C, panel i–iii). To further determine the extent of microstructural changes mediated in H9c2 cardiomyocytes treated with cocaine at higher concentrations, cells were exposed to 50 µg/mL and 100 µg/mL cocaine treatments and visualised using SEM (Figure 8). SEM reconnaissance revealed the extensive presence of membrane associated blebbing in and around H9c2 cardiomyocytes with active separation of formed blebs from the cellular periphery following 50 µg/mL treatments of cocaine (Figure 8, panel i–iii). Comparatively, treatments with 100 µg/mL cocaine induced significant cellular damage, associated with the loss of intracellular integrity, intercellular membrane outgrowth and nuclei alterations (Figure 8, panel i,ii). Also apparent was the mediation of cardiomyocyte cell condensation with cellular perforation surrounding the nuclei and its separation from the cell body (Figure 8, panel iii). These results highlight the role of cocaine in mediating microstructural alterations associated with functional activity and cytotoxicity in cardiomyocytes.

## 3. Discussion

The present study sought to investigate the phenotypic consequence of cocaine in cardiomyocyte cell lines derived from BD1Z rat cardiac tissue. These cell lines have previously been shown to be effective in elucidating the toxic effects of drugs, along with myocyte damage and cytoskeletal rearrangements associated with ABPs [9,10,11]. Though previous studies have examined the cytotoxicity and functional implications of cocaine, they have primarily concentrated on neuronal properties involved in mechanisms of addiction, with the majority implementing serum free or reduced serum in vitro conditions, in vivo models, case, and prospective studies [4,12,13,14,15]. To our knowledge, only four research articles have utilised the H9c2 cardiomyocyte cell line in cocaine-based studies relating to cardiotoxicity, all of which employed the use of serum conditions and investigated specific regulatory functions relating to cardiotoxicity [10,16,17,18]. Importantly, studies into the regulatory and cytotoxic functions mediated by cocaine in H9c2 cardiomyocytes permits an understanding into its role without the possible attribution of sympathomimetic effects, owing to the lack of neurons and hormonal influences in this model [19]. 

The cocaine concentration chosen by this study was 1 µg/mL, compared to previous literature relating to both cardiac and non-cardiac in vitro models this dosage lies within the plasma ranges conventionally observed in recreational drug users [12,19,20,21,22,23,24]. The cocaine treatment selected in the present study was also considered in relation to reports that have shown insufflation of 1.5 mg/kg (equating to 105 mg/70 kg person) results in peak urine concentrations of 0.3 µg/mL with post-mortem results highlighting mortality in 100 cases at cocaine concentrations of 9.8 1 µg/mL) and other studies demonstrating cocaine induced apoptosis between 0.1–1.0 µmol/mL treatments [25,26,27]. Moreover, a study by Mittleman and Wetli [28] showed the average plasma concentration of cocaine in a deceased cohort was 6.2 mg/L a level comparatively higher than the concentration employed in this study.

Our result demonstrated that in response to cocaine concentrations of 10 µg/mL H9c2 cardiomyocytes underwent enhanced loss of cellular compaction and cell-cell integrity, common features of post-natal cardiac hypertrophy while doses of 1 µg/mL, appeared to cause increased cell polarity [29]. Studies performed by Badisa et al. [24] suggested that the cell types examined are more inclined to withstand greater cocaine concertation, however, the present investigation indicates cardiomyocytes are more sensitive to higher cocaine concentration exposures. This finding corroborates Wu et al. [16], which demonstrated cocaine concentrations between 1–100 µM abrogate potassium channel activity which in turn alters functional regulation of cardiac cells associated with cardiac arrhythmias, a known implication of cocaine induced cardiac dysregulation [11,30]. SEM analysis indicated that treatments at 50 µg/mL cocaine enhanced the formation of cell periphery and surface blebbing with significant reductions in cellular outgrowths and concomitant loss of spindle-to-satellite architecture. The cytotoxicity of cocaine in cardiac cells has previously been shown to occur via apoptotic mechanisms. An earlier study by Welder et al. [31] demonstrated the toxic effects of cocaine in primary cultures of rat cardiac muscle and reported that treatments of 1 x 10^-3^ M (1000 µM) mediated enhanced vacuolisation, granulation, pseudopodia formation and lactate dehydrogenase release, the latter of which has been shown to represent a clinical signature for cocaine induced cardiotoxicity and apoptotic cell damage [32,33,34]. Utilising fetal rat myocardial cells another study by Xiao et al. [34] demonstrated by phase contrast microscopy the lobulated, rounded and reduced size of cells following cocaine treatment, morphological characteristics of which agree with our findings. Compared to the literature which has concentrated on other cardiac in-vitro models and have reported cocaine induced cardiac cell apoptosis, no current study cites the involvement of surface and membrane blebbing in fetal cell lines or primary cells, possibly suggesting its attribution to cardiomyocyte death in embryonic heart tissue following in-utero cocaine exposure [35]. Our findings of cytotoxicity at 5 µg/mL doses fall within the cited range of cocaine mediated apoptosis in cardiac cells, as active drug abusers’ serum cocaine levels are more likely to be > 100 µM [36]. Therefore, the finding of the current study that cocaine treatments in foetal cardiomyocytes mediates the potential morphological appearance of apoptotic cells associated with membrane and surface remodelling, in addition to the loss of cellular architecture warrants its pathophysiological relevance to cardiotoxicity induced drug abuse. 

The in vitro scratch assay mimics cellular migration during wound healing in vivo, a responsive mechanism to pathological injury involving two-dimensional collective cell motility. More specifically, the migration investigated in the present study probed the effect of cocaine on sheet migration of cardiomyocytes, a mechanism involved in tissue injury and embryonic morphogenesis [37]. The findings of the current study suggest that the exposure of cocaine to cardiomyocytes increased migratory activity towards the stimulated wound. By analysing the number of cells and their percentage closure of stimulated wounds, this study highlights for the first time the mitogenic properties of cocaine on cardiomyocytes. It should be noted that this increase in cell migration is not accompanied by increased cell proliferation, indicating a change in cytoskeletal dynamics with limited evidence of regeneration. Significantly, the wound closure of cardiomyocytes treated with cocaine following 24-h of exposure is suggestive of the capacity of these cells involved in pathological injury. A study by Itou et al. [38] examined the reparative nature of the heart following injury and reported cardiomyocyte migration as an essential factor involved in cardiac regenerative capacity. Typically, the cardiac tissue displays limited resolution following injury; therefore, the potential for cocaine mediated wound healing as observed in this study is relevant to reparative events associated with cardiac fibrosis following continual myocardial infarctions observed in habitual abusers [2,39]. A study by Maceira et al. [4] demonstrated focal myocardial fibrosis in chronic cocaine abusers which emphasises the role cardiomyocytes may have in regulating repair and pathology following chronic cocaine exposures. 

The present study can attribute with confidence the microstructural significance associated with cell migration in cardiomyocytes treated with 1 µg/mL. Scanning electron microscopy images indicate that cocaine treatments significantly alter membrane polarity associated with a migratory phenotype, with the mediation of altered membrane dynamics, cell-substrate adhesion and membrane protrusions associated with pseudopodia formation. Also present was cell body contraction and membrane folding at cellular extremities, alongside the presence of attachment and membrane extensions at the rear and front of cell. To fully examine the role of cocaine as a migratory mediator, the present study investigated the expressional dynamics of several key ABPs. Cofilin is the master regulator of non-equilibrium actin filamentous assembly/disassembly and has been shown to mediate significant alterations to cellular phenotypes associated with fibrosis, with recent studies such as Lee et al. [6] demonstrating the role of the Rho/ROCK/cofilin pathway in myocardial fibrosis following in vivo induced infarctions. Owing to the response of tissues to pathological injury and the consequent reorganisation of the actin cytoskeleton, a number of studies have demonstrated the dynamic characteristic of cofilin expression in pathological injuries associated with upregulated fibrotic development and dysregulated wound healing mechanisms [40,41,42]. Therefore, in the context of cardiac injuries such as myocardial infarctions, commonly thought of as a dysregulated wound healing mechanism, our results indicate the early upregulation (dephosphorylation) of cofilin expression following cardiomyocyte cocaine treatment. 

These results corroborate previous work investigating the structural and behavioural plasticity of drug addiction within the NAc. Several authors have investigated the altered expression of cofilin and the dynamic change associated with LIMK regulatory activity in relation to acute and repeated withdrawal from cocaine in in vivo models. Toda et al. [43]; Wang et al. [5] and Calipari et al. [15] also highlighted dephosphorylation of cofilin subsequent to repeated cocaine exposure and suggested the enhanced spine density observed was the result of lamellipodia-like protein complexes as oppose to a filopodia profile of ABP expression. The data presented by in-silicio binding studies also demonstrated the interactive nature of cocaine and BZE to common ligand binding sites found in cytoskeletal regulators and signal transduction modulators. Using key upstream regulators involved in mediating downstream cytoskeletal effects examined in wet laboratory conditions, in silico docking demonstrated the potential role cocaine may have in mediating binding capacity to ROCK and LIMK. Furthermore, by modelling cocaine and its metabolite to the ATP binding pocket of ROCK and LIMK cytoskeletal modulators, the docking studies revealed that BZE obtained a more energetically favourable affinity for ROCK when compared to cocaine and that either ligand (cocaine or BZE) may be energetically permissive to regulate LIMK activity. While LC-MS studies have shown that in in vitro settings H9c2 cardiomyocytes metabolises cocaine into BZE and that within 24 h of cocaine treatment BZE is the predominant drug present in the cell media. This represents a significant decrease in metabolism time compared to in vivo settings where cocaine is typically converted to BZE within one hour. In this way it is possible that some of the later alterations in cytoskeletal dynamics may be driven, in part, by BZE because of its metabolism from the parent drug. Taken together, with the possibility of greater cocaine-sigma-1 receptor interactions, these findings may indicate a signalling network in which the upstream activity of the sigma-1 receptor in response to cocaine functions to connect and induce the activity of key cytoskeletal regulators, as suggested by Yao et al. [44], which in turn is further potentiated by the metabolite BZE. 

The current study examined changes in ERM and VASP ABPs, both of which are of significance to the pathologies involving fibrotic mechanisms [45,46]. The evolutionary conserved ERM ABPs have been reported to mediate epithelial cell architecture, integrity, polarity and morphogenesis in addition to contributing to cardiomyopathy and contractile dysfunction in post-natal hearts [47,48]. We demonstrated enhanced phosphorylation of ERM proteins in cardiomyocytes following cocaine exposure. Only one other study in the literature has demonstrated cocaine regulated changes in ERM expressional dynamics. Kim et al. [49] reported a reduction in ERM phosphorylation in rat brain tissues. Our results suggest VASP regulated cardiomyocyte activity was not affected by cocaine. Several authors in recent years have demonstrated the involvement of specific VASP phosphorylation sites to cardiac dysregulation with, S157 and S239 phosphorylation mediating cardiac hypertrophy and Rac-1 mediated actin cytoskeletal remodelling during pulmonary endothelial cell barrier formation [50,51]. While other studies have suggested a role for VASP in filipodia formation, in this study VASP expression was not linked to early cytoskeletal changes following cocaine treatment. However, the finding of enhanced ERK activity during the earlier time points corresponds with literature in which several authors have demonstrated the localisation of ERK to protrusion and adhesion sites in motile cells responding to extracellular cues [52,53]. A study by Mendoza et al. [54] investigated the regulatory mechanisms of ERK during cellular motility and demonstrated its co-localisation with WRC (WAVE regulatory complex), a protein complex of the Wiskott–Aldrich syndrome protein (WASP) family that functions to mediate cytoskeletal dynamics via Arp2/3.

Our results indicate that cocaine increased adhesion in a dose related manner, with adhesion increased in cardiomyocytes exposed to low dose cocaine concentrations (0.1, 0.25, and 0.5 µg/mL) when compared to 1 µg/mL treatments. Our results also showed a decrease in cardiomyocytic integrin-β3 expression in response to cocaine. However, though the results indicated a greater degree of cell-substrate adhesion following 0.1 µg/mL exposures, this remains within conventional plasma ranges for cocaine concentrations observed in recreational drug users. Therefore, to further clarify the possibility for the induction of cell-substrate adhesion by cocaine at 1 µg/mL and to understand the possible mediators involved in this process, preliminary expressional analysis for the key integrin subtype integrin-β3 was undertaken. In the context of pathophysiology, studies by Okada et al. [55] demonstrated the cardioprotective role of integrins during ischemia, with investigations by Shewchuk et al. [56] implicating integrin-β3 with protective mechanisms against oxidative stress induced apoptosis in cardiomyocytes, an early event during cocaine mediated cardiac toxicity [57]. In the current study, integrin-β3 was downregulated in response to cocaine suggesting a possible loss of this protective process.

This study has demonstrated the migratory and cell-substrate adhesion properties of cocaine, phenotypes which are both upregulated during inflammatory and fibrotic processes that govern cardiac dysregulations including cocaine induced coronary atherosclerotic plaque formation, a primary pathology of acute myocardial infarction [58,59,60]. Moreover, the results demonstrate novel and compelling data to which further investigations may be mapped towards cardiac toxicity and the elucidation of its mechanisms governing cardiomyocyte death as a factor for the induction of cardiac fibroblasts during injury induced tissue remodelling. In the context of fibrotic mechanisms, the alterations mediated by cocaine treatments in relation to cardiomyocytes presented in this study are suggestive for early responses to tissue remodelling and pathological injury. A significant literature base exists which reports myocardial dysregulation following cocaine abuse, with myocardial infarction induced by vascular spasm and platelet aggregation and coronary-artery vasoconstriction being the most prevalent [61,62]. Cocaine induced acute myocardial infarction and the subsequent cellular dysregulation is associated with the loss of cardiomyocytes and the induction of inflammatory responses and recruitment of cardiac fibroblasts [2]. This in turn results in activation of fibroblasts into a myofibroblast phenotype and induces the formation of a non-contractile ECM protein rich tissue. In the short-term this permits organ function, however, there is a decline in cardiac performance and the heart is rendered susceptible to subsequent cardiac dysfunction. Though this cascade of events stands well established, the involvement to which cocaine represents in mediating its progression remains unknown, particularly relating to cytoskeletal change. Further work in relation to the possible role of cocaine induced cardiomyocyte-fibroblast communication in the context of pathology may shed further light on the cardiac dysregulating potential of cocaine.

## 4. Materials and Methods

### 4.1. H9c2 Embryonic Cardiomyocyte Cell Lines

H9c2 embryonic cardiomyocyte (Lonza) cell sub-clones, derived from the parental clone cell line embryonic BD1X rat heart tissue was used in this study. The H9c2 cardiomyocyte cell line has widely been utilised for cardiotoxicity studies for the analysis of morphological characteristics, as changes in cellular activity resemble immature embryonic cardiomyocytes with many signalling pathways conserved for their differentiation into mature myocardial cells. Furthermore, the H9c2 cardiomyocyte cell line has commonly been used in various studies relating to drug toxicity pathways (American Type Culture Collection (ATCC)) and to this end provided a viable in vitro model [63]. Cells were either sub-cultured into 75 cm^2^ sterile flasks at the required culture ratio of either 1:5 or 1:10 or seeded at adequate densities into six-well or 96-well tissue culture plates. Initial cell starting passage was between four and six, with all experiments performed between passages 8–12.

### 4.2. Chemicals and Reagents

The following chemical and reagents were used in the experimental protocols described below. Cocaine 1mg/mL (Cerilliant, Round Rock, Texas USA) was used for all treatments and diluted in cell media. Cell media was comprised of Dulbecco’s modified Eagle medium (DMEM) (Sigma-Aldrich, USA) supplemented with 10% Foetal Bovine Serum (FBS), 1% (100 U/mL) penicillin and (100µg/mL) streptomycin, (all Sigma-Aldrich, St Louis, MO, USA). Dulbecco’s phosphate-buffer saline (DPBS) and dimethethyl sulfoxide (DMSO) (both Sigma-Aldrich, USA) was added during cryopreservation. For cell proliferation studies, a BrdU incorporation assay (Merck Millipore, USA) kit was used. Cell lysis was performed using radioimmunoprecipitation assay (RIPA) buffer (Sigma-Aldrich, USA) containing protease inhibitor cocktail (1:100) (Abcam, Cambridge, UK), phosphatase inhibitor cocktail (1:100) (Sigma-Aldrich, USA) and 1 mM phenylmethanesulfonyl fluoride solution (Sigma-Aldrich, USA). Protein quantification was carried out using a Bradford reagent (Sigma-Aldrich, USA), cell lysates, NuPAGE LDS Sample Buffer 4X (Thermo Fisher Scientific, Hampton, New Hampshire, USA) containing 2% (*w*/*v*) dithiothreitol (DTT) (Thermo Fisher Scientific) Primary Antibodies for immunoblotting studies are detailed in Section 4.4. For cell adhesion assay, Fibronectin (FN) and bovine-serum albumin (BSA) (both Sigma-Aldrich, USA) diluted in PBS were used for positive and negative controls, respectively. For LC-MS analysis, organic mobile phase was HPLC grade Acetonitrile and aqueous was HPLC grade water (both VWR chemicals), with 0.1% formic acid (Sigma-Aldrich, USA) added to both mobile phases and 0.01g/L of ammonium acetate added to organic solution only. Benzoylecgonine, D8-Benzoylecgonine and D3 Cocaine were used as reference standards (Cerilliant,). Microscopy fixation was carried out using 2.5% (*v*/*v*) glutaraldehyde solution (Sigma-Aldrich, USA). 1% (*w*/*v*) osmium tetraoxide (OsO4) (Agar Scientific). Dehydration was performed using graded ethanol solutions (VWR Chemicals, Radnor, Pennsylvania, USA) and hexamethyledisilizane (Sigma-Aldrich, USA). 

### 4.3. Cell Viability and Toxicity Assay

Cellular growth and gross morphological responses of H9c2 cardiomyocyte cells to differing concentrations of cocaine were analysed using the IncuCyte Zoom^®^ integrated confluency algorithm. H9c2 cardiomyocytes were seeded at a density of 6.0 × 10^3^ cells/well in complete growth medium and allowed to adhere prior to drug exposure for 24 h to reach 50–60% confluency. Confluent cells were treated with three different concentrations (10, 50, 100 µg/mL) of cocaine diluted in complete growth medium. Cells were incubated for 72-h and images acquired continuously at two-hour intervals under phase contrast using the IncuCyte Zoom^®^ (S2, Essen Bioscience, Ann Arbour, Michigan, USA) system from three independent experiments, and statistically analysed using the IBM SPSS 24™ statistical package. Statistical comparison was performed by a one-way ANOVA followed by the post hoc Dunnent’s comparison test, * *p* < 0.05 for each group vs control. 

### 4.4. Scratch Wound Assay

To model chronic drug exposure as a mechanism for determining the potential migratory effect of cocaine on H9c2 cardiomyocytes following injury, cells were grown in complete growth medium containing low dose cocaine concentrations (1 µg/mL). H9c2 cardiomyocyte cells were cultured (3.0 × 10^5^ cells/well) and grown to confluency in clear sterile 6-well culture plates (Corning Costar^®^) in complete growth medium supplemented with 1 g/mL cocaine for 24 h. In these studies, cells treated with complete growth medium alone served as controls with medium replenished concurrently with treated samples. H9c2 cells were cultured in high glucose (4500 mg/L) Dulbecco’s modified Eagle medium (DMEM) (Sigma- Aldrich, USA) supplemented with 10% Foetal Bovine Serum (FBS), 1% (100 U/mL) penicillin, (100 g/mL) streptomycin, L-glutamine, sodium bicarbonate and 100 mM sodium pyruvate (3.0 × 10^5^ cells/well) to confluency. Cells were treated with 1 µg/mL of cocaine for 24 h. Formation of a monolayer was visualised at two-hour intervals using the IncuCyte Zoom^®^ system during the initial 24-h growth period. Upon reaching confluency (70–80%), cells were wounded using a 200 μL sterilised plastic pipette and washed once with warm DPBS. Stimulated wounded areas were visualised and images captured using the AMG Evos FL™ inverted microscope. Wound area measurements taken from five selected fields were taken using the ImageJ Image Processing and Analysis software™, and percentage wound closure determined. Measurements were statistically analysed from three independent experiments using the IBM SPSS Statistics 24™ software package. Statistical comparison was performed by a two-tailed Students unpaired t-test, *p < 0.05 for treated vs. control. 

### 4.5. Immunoblotting

After cocaine stimulation, cells were lysed with RIPA buffer in the presence of protease inhibitor cocktail and phosphatase inhibitor cocktail 2. Proteins were fractioned using 10% or 12% SDS–PAGE and analysed using immunoblotting with the following antibodies: phosphorylated at Ser 3, ERM phosphorylated at Thr-567, Thr-564, and Thr-558,VASP phosphorylated at Ser-239, ERK1-p44 phosphorylated at Thr-202/Tyr204 and ERK2-p42 Thr-185/Try-187, β3-Integrin, β-actin. Secondary antibodies were horseradish peroxidase labelled anti mouse or anti rabbit. All antibodies were purchased from Cell Signaling Technology and were used at a dilution of 1:1000 for primary antibodies and 1:500 for secondary antibodies. Densitometry analysis on the bands was performed using the Image J Image Processing and Analysis software™ and the data was normalised to total protein levels.

### 4.6. Cell Proliferation Assay

Cell proliferation was evaluated in response to 1 g/mL of cocaine for 24 h, by the bromo-2’-deoxy-uridine (BrdU) incorporation assay. Following removal of the BrdU label, cells were fixed and denatured with BrdU Fixative/Denaturing solution and labelled with 100 L/well anti-BrdU conjugated antibody (1:100). The reaction was detected with chromogenic peroxidase substrate, tetra-methylbenzidine (TMB) following the addition of the pre-supplied stop solution with absorbance measured using a Tecan Infinite M200 PRO plate reader at OD 450.

### 4.7. In-Vitro Cell-Substrate Adhesion Assay

To ascertain the potential role cocaine may have in cell mediated substrate interactions, cell-substrate adhesion experiments were performed using extracellular environments containing several cocaine concentrations with fibronectin (FN) as a positive control and DPBS as a negative control. FN (Sigma-Aldrich, USA) and cocaine stocks were prepared fresh to working concentrations (1 g/mL) diluted in DPBS (Sigma, USA) void of calcium and magnesium divalent ions. Ninety-six transparent plates were coated with FN, DPBS or cocaine stocks of 0.1, 0.25, 0.5, or 1 g/mL. Fibronectin was used as a positive control and DPBS served as a negative control. The 96 plates were coated with FN, DPBS and cocaine stocks and incubated overnight at 4 °C. Subsequently, H9C2 cardiomyocytes were cultured and counted using the trypan blue dye exclusion assay. Adherent cells were washed once with sterile filtered DPBS containing calcium chloride, 0.0133% (*w*/*v*) and 0.01% (*w*/*v*) magnesium chloride inorganic salts (Sigma-Aldrich, USA) and fixed with 4% paraformaldehyde at room temperature for 15 min. Following fixation, cultured wells were washed with DPBS and labelled for 15 min with 0.5% crystal violet dye prepared in 100% ethanol water solution overnight. Subsequently, cells were lysed with 1% sodium dodecylsulphate (SDS) (Sigma-Aldrich, USA) before absorbance at OD 570 was measured. Cell adhesion assays were performed from three independent experiments and statically analysed using the IBM SPSS Statistic 24 software package (Armonk, NY, USA). 

### 4.8. LC-MS Analysis

Cell were treated with 1 µg/mL of cocaine and media suspension was then removed at specific timepoints between 10 min and 24 h. Samples were spiked with deuterated internal standard for cocaine and BZE. Samples, calibrators, blanks, and quality control (QC) standards had a starting volume of 1 mL of cell media in conical centrifuge tubes (Fisher Scientific). After addition of drug standards, samples were sonicated for 15 min and buffered in 0.1 M phosphate buffer pH 7. Samples were centrifuged at 2500 RCF for 10 min and the supernatant passed through an Isolute HCX 130 mg/10mL cartridges (Biotage) for extraction. Samples were eluted in an organic solvent and ammonia mix (10 mL of propan-2-ol, 1 mL ammonia, 39 mL of dichloromethane) before evaporation under nitrogen flow at 40 °C. Samples were reconstituted in 160 µL of 50/50 of mobile phase A (H_2_O 0.1% formic acid) and mobile phase B (acetonitrile 0.1% formic acid).

### 4.9. Scanning Electron Microscopy

SEM was used to visualise morphological changes in H9c2 cardiomyocyte cells in response to cocaine at concentrations of 1, 5 & 10 g/mL. Cells were fixed using 2.5% (*v*/*v*) glutaraldehyde solution diluted in 0.1M phosphate buffer. Following fixation, cells were washed in DPBS and exposed to secondary fixation containing 1% (*w*/*v*) osmium tetraoxide (OsO_4_). Cells were dehydrated in graded ethanol concentrations (50, 70, 80, 90, 95 100% (*v*/*v*)) and immersed in hexamethyledisilizane, then mounted onto glass slides and exposed to gold palladium alloy sputter coating (Polaron Range SC7640). Coated samples were subsequently examined using the Zeiss™ EV050 scanning electron microscope.

### 4.10. In-Silico Bioinformatic Docking Studies

Using the software Scigress Fujitsu™, various signalling modulators involved in mediating ROCK driven cytoskeletal dynamics were analysed, with the sigma-1 receptor and dopamine reuptake transporter (DAT) serving as positive controls for known cocaine binding. Molecular modelling and drug ligand-docking studies were performed using software Scigress Fujitsu™. Relevant molecular structures corresponding to cocaine and its metabolite BZE were programmed into the software prior to modelling. FASTA format peptide sequences were subsequently imported into the SWISS-MODEL protein structure homology-modelling server for the generation of three-dimensional protein structural models derived from X-ray diffraction and NMR studies. Structural coverage levels were used to evaluate protein domain specific sequences. Generated models were then imported under PDB format into the Scigress Fujitsu™ software and managed according to specific domains of interest. Docking sites of interest and ligands were imported to determine binding energy output in kilocalorie/mole for specified docking sites.

## Abbreviations

ABP	Actin binding protein
BrdU	Bromo-2′-deoxy-uridine
BZE	Benzoylecgonine
DPBS	Dulbecco’s Phosphate Buffered-Saline
DMEM	Dulbecco’s modified eagle medium
ECM	Extracellular matrix
ERK	Extracellular regulated kinase
ERM	Exrin Radixin Moesin
FASTA	Format DNA and Protein Sequence Alignment
FBS	Fetal bovine serum
FN	Fibronectin
LC-MS	Liquid Chromatography Mass Spectrometry
LIMK	LIM-kinase
MAPK	Mitogen-Activated Protein Kinase
NAc	Nucleus accumbens
RIPA	Radioimmunoprecipitation assay
ROCK	Rho- associated kinase
SEM	Scanning Electron Microscopy
VASP	Vasodilator stimulated phosphoprotein
